# High-resolution mapping of mitotic DNA synthesis regions and common fragile sites in the human genome through direct sequencing

**DOI:** 10.1038/s41422-020-0358-x

**Published:** 2020-06-19

**Authors:** Morgane Macheret, Rahul Bhowmick, Katarzyna Sobkowiak, Laura Padayachy, Jonathan Mailler, Ian D. Hickson, Thanos D. Halazonetis

**Affiliations:** 1 Department of Molecular Biology, University of Geneva, 1205 Geneva, Switzerland; 2 Center for Chromosome Stability and Center for Healthy Aging, Department of Cellular and Molecular Medicine, University of Copenhagen, Blegdamsvej 3B, 2200 Copenhagen N, Denmark

**Keywords:** Cancer, DNA damage and repair

## Abstract

DNA replication stress, a feature of human cancers, often leads to instability at specific genomic loci, such as the common fragile sites (CFSs). Cells experiencing DNA replication stress may also exhibit mitotic DNA synthesis (MiDAS). To understand the physiological function of MiDAS and its relationship to CFSs, we mapped, at high resolution, the genomic sites of MiDAS in cells treated with the DNA polymerase inhibitor aphidicolin. Sites of MiDAS were evident as well-defined peaks that were largely conserved between cell lines and encompassed all known CFSs. The MiDAS peaks mapped within large, transcribed, origin-poor genomic regions. In cells that had been treated with aphidicolin, these regions remained unreplicated even in late S phase; MiDAS then served to complete their replication after the cells entered mitosis. Interestingly, leading and lagging strand synthesis were uncoupled in MiDAS, consistent with MiDAS being a form of break-induced replication, a repair mechanism for collapsed DNA replication forks. Our results provide a better understanding of the mechanisms leading to genomic instability at CFSs and in cancer cells.

## Introduction

The accurate duplication of the genome is fundamental for cell viability. In eukaryotes, DNA replication initiates at specific genomic locations called replication origins. Origin firing generates two replication forks that advance bidirectionally along the parental DNA template until they each encounter a converging replication fork.^1^ Although, in principle, the above process can ensure complete replication of the genome, several obstacles can be encountered that have the potential to arrest or perturb ongoing fork progression. These include atypical structures formed in the DNA template, such as hairpins and G-quadruplexes, or encounters with the machineries carrying out other DNA metabolic processes, such as transcription.^2–5^ Curiously, many eukaryotic genomes harbor evolutionarily conserved regions that appear intrinsically difficult to replicate. The most extensively studied of these regions are the so-called common fragile sites (CFSs).^6–11^ These sites are of broad interest, because they very frequently correspond to sites of genomic rearrangements in human cancers; indeed, six of the ten most common loci for recurrent focal deletions in human cancers lie within CFSs.^12^

CFSs, so named because they are present in the genome of all individuals, are expressed as breaks or gaps on mitotic chromosomes of cells treated with the DNA polymerase inhibitor aphidicolin.^6^ Around 86 aphidicolin-induced CFSs have been mapped at low resolution by cytogenetic methods.^9,13^ A handful of these have also been mapped at higher resolution by in situ hybridization analysis of chromosomes with YAC and BAC probes.^9,14–18^ Many CFSs map within large genes^14,15,19,20^ that are transcriptionally active^13^ and that replicate in late S phase.^21–23^ Additionally, some CFSs correspond to regions with a low replication origin density,^24^ while others may have a normal origin density, but lack dormant origins that can fire under conditions of DNA replication stress.^25^

What makes CFSs sensitive to DNA replication stress is still a matter of debate.^9,11,26–29^ The fragile loci visible on metaphase chromosomes may correspond to regions of the genome that have failed to complete replication prior to mitotic entry. One attractive model proposes that CFSs require a substantial time to replicate because they correspond to large, late-replicating, origin-poor genomic regions.^24^ An alternative model, based on the observation that CFSs often map within transcribed genes,^13,17,20,24,30,31^ proposes that the transcription machinery either induces fork collapse or that it displaces origin recognition complexes preventing these origins from firing. In either case, the result would be a failure to complete replication of the locus during interphase. Yet another possibility is that the DNA sequences present within the CFSs may be inherently difficult to replicate due to their propensity to adopt atypical secondary structures.^16,32–36^

Interestingly, treatment of cells with aphidicolin leads not only to breaks and gaps on mitotic chromosomes, but also to foci of nascent DNA synthesis that decorate these chromosomes.^37^ These foci represent sites of so-called mitotic DNA synthesis (MiDAS), and generally colocalize with FANCD2 foci and with ultrafine anaphase bridges, which mark the chromosomal positions of CFSs in cells exposed to aphidicolin.^37–39^

MiDAS shares features with break-induced DNA replication (BIR), a homologous recombination-based pathway for repair and restart of collapsed DNA replication forks.^40–42^ MiDAS is dependent on genes that function in BIR, such as *POLD3*, *RAD52*, *FANCD2* and *SLX4*.^37,43–48^ In addition, some, but not all, of the MiDAS foci on mitotic chromosomes involve only one of the two sister chromatids, which is consistent with conservative DNA replication, a feature of BIR.^37,49–51^ Nevertheless, the precise biological function of MiDAS is unclear. MiDAS might help complete replication of genomic loci that had not been replicated prior to mitotic entry. Alternatively, MiDAS might be involved in repair of DNA double-strand breaks and other forms of DNA damage in mitotic cells.

Here, we studied aphidicolin-treated cells using EdUseq^52^ to map the genomic regions that remain under-replicated prior to mitotic entry and the genomic sites undergoing MiDAS. Our analysis revealed that the main function of MiDAS is to help complete replication of the genome. The genomic regions replicated by MiDAS encompass all known CFSs, as well as a repertoire of other CFS-like sites, all of which are now mapped at high resolution. We also provide evidence supporting the hypothesis that MiDAS is mediated by BIR, as well as mechanistic insights that help explain why CFSs are prone to genomic instability in human cancers and in cells treated with DNA replication inhibitors.

## Results

### MiDAS initiates from the borders of replicated/unreplicated genomic regions

**Fig. 1:**
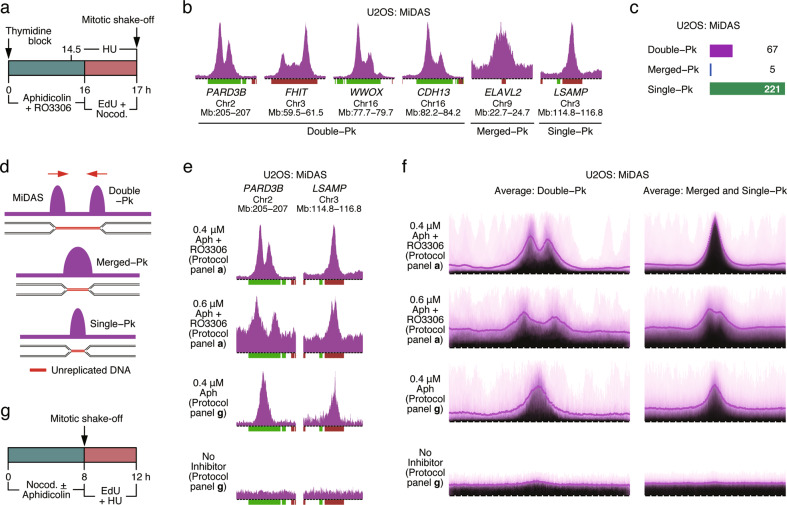
Characterization of MiDAS sites in U2OS cells. **a** Experimental outline of the protocol to monitor MiDAS. Nocod, nocodazole; HU, hydroxyurea. **b** Representative examples of the three types of MiDAS regions. MiDAS signal (sigma values) is shown in purple; genes are displayed below and are colored green or red (forward or reverse direction of transcription, respectively). Chromosome number and genomic coordinates of the regions shown are listed below the gene names. Peak heights in sigma units: *PARD3B*, 109.6; *FHIT*, 393.9; *WWOX*, 365.1; *CDH13*, 242.5; *ELAVL2*, 46.7; *LSAMP*, 84.6. Bin resolution, 10 kb; ruler scale, 100 kb; Pk, peak. **c** Number of MiDAS regions of each peak type. **d** Proposed mechanism for the existence of three distinct MiDAS peak types. The arrows indicate the proposed direction of fork movement. **e**, **f** MiDAS following different permutations of the MiDAS protocol. **e** MiDAS at specific genomic sites. **f** Average MiDAS signal of all double-peak regions (left panels; span of genomic region, 2.9 Mb) and all merged- and single-peak regions (right panels; span of genomic region, 2.3 Mb). From top to bottom: standard protocol (panel **a**; 0.4 µM aphidicolin); standard protocol with increased dose of aphidicolin (0.6 µM); alternative protocol (panel **g**) with or without addition of aphidicolin (0.4 µM). Aph, aphidicolin. **g** Experimental outline of the alternative protocol to monitor MiDAS.

We established a protocol to sequence the genomic regions undergoing MiDAS (Fig. 1a). Briefly, cells were synchronized with thymidine at the G1/S boundary and then released into S phase in the presence of aphidicolin and RO3306. Aphidicolin slows down DNA replication, while RO3306, a CDK1 inhibitor, prevents entry into mitosis. Sixteen hours later, aphidicolin and RO3306 were removed, and EdU and nocodazole were added to the media. EdU labels nascent DNA, while nocodazole arrests cells in prometaphase and prevents mitotic exit. One hour later, the prometaphase-arrested cells were collected by mitotic shake-off and their EdU-labeled genomic DNA was affinity-purified and subjected to high throughput sequencing (Fig. 1a). At the time that the mitotic cells were harvested, a small fraction of the cells in the population were still in S phase, and hence were incorporating very high levels of EdU that could contaminate the EdU signal from the mitotic cells (Supplementary information, Fig. S1a). To avoid this, hydroxyurea (HU) was added to the medium 2.5 h prior to the mitotic shake-off (Fig. 1a). HU reduces the nucleotide pool to levels unable to support DNA synthesis of cells in S phase, but has little impact on the low levels of DNA synthesis occurring in mitotic cells (see below).

First, we examined MiDAS in U2OS human osteosarcoma cells (Fig. 1b). Using a custom script, a total of 293 MiDAS regions were identified and these could be divided into three types: double-peak (*n* = 67), merged-peak (*n* = 5) and single-peak (*n* = 221) (Fig. 1c; Supplementary information, Fig. S1b). Examples of double-peak MiDAS regions mapping to the genes *PARD3B*, *FHIT*, *WWOX* and *CDH13*, respectively, are shown in Fig. 1b. The merged-peak MiDAS regions resembled double-peak regions, except that there was little or no space between the two peaks (Fig. 1b, region mapping to the *ELAVL2* gene), whereas the single-peak MiDAS regions had a peak width similar to that of one peak of the double-peak regions (Fig. 1b, region mapping to the *LSAMP* gene).

One interpretation for the existence of three types of MiDAS regions is that DNA synthesis in mitosis initiates from collapsed DNA replication forks present at the border of the replicated/unreplicated genomic regions (Fig. 1d). According to this interpretation, when the unreplicated genomic region is above a certain size, the two peaks of nascent DNA synthesis at each end of the unreplicated region would be well-separated (Fig. 1d, double-peak region). For shorter unreplicated genomic regions, the two peaks would be less well-resolved, resulting in little or no space between them (Fig. 1d, merged-peak), or may completely overlap and appear as a single-peak (Fig. 1d, single-peak).

To explore the above interpretation, we performed various permutations of the MiDAS protocol. In the first permutation, we increased the dose of aphidicolin during S phase from 0.4 to 0.6 μM (Fig. 1a). We anticipated that this would lead to an increase in the length of the unreplicated genomic regions, a prediction confirmed by inspection of individual MiDAS regions (Fig. 1e) and the genome-wide averages of all MiDAS regions (Fig. 1f). Under these conditions, most of the single-peak MiDAS regions were transformed into double-peak regions (Fig. 1e, f). In a second permutation, we modified the MiDAS protocol to label nascent DNA in cells that had already advanced into prophase/prometaphase prior to the addition of EdU. In this second protocol, unsynchronized U2OS cells were treated with aphidicolin and nocodazole for 8 h, and then the prophase/prometaphase cells were isolated and EdU and HU were added to the media (Fig. 1g). Under these conditions, all double-peak MiDAS regions were transformed into single-peak regions (Fig. 1e, f). In a variation of this second protocol, when no aphidicolin was added to the cells, little or no MiDAS was observed (Fig. 1e, f), suggesting that MiDAS is observed mostly when DNA replication is perturbed and only rarely under normal growth conditions. Finally, since the width of the MiDAS regions varied according to the experimental protocol used, it appears that there are no site-specific roadblocks to DNA replication within the MiDAS regions.

**Fig. 2:**
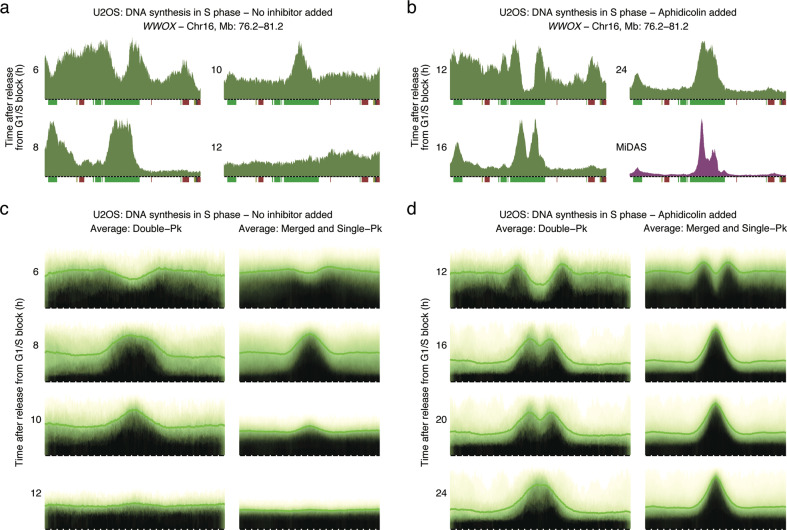
MiDAS peaks correspond to the borders of replicated/unreplicated genomic regions. **a**, **b** Replication signal (green) around the *WWOX* gene at the indicated times after release from a thymidine block in the absence (**a**) or presence (**b**) of 0.4 µM aphidicolin. The MiDAS signal for the same genomic site is shown in purple in **b**. Bin resolution and ruler scale are as in Fig. 1b. **c**, **d** Genome-wide average replication signal at double-peak (left panels; span of genomic region, 2.9 Mb) and merged- and single-peak (right panels; span of genomic region, 2.3 Mb) MiDAS regions at the indicated times after release from a thymidine block in the absence (**c**) or presence (**d**) of 0.4 µM aphidicolin. Pk, peak.

To further strengthen the model that MiDAS corresponds to regions of the genome that remain under-replicated prior to mitotic entry, we monitored DNA replication during S phase. The cells were released from a thymidine block and allowed to progress through S phase in the presence or absence of low dose aphidicolin and/or RO3306. At various timepoints after release from the thymidine block, EdU was added to the medium and 30 min later the cells were harvested and the nascent DNA sequenced. Using the genomic region mapping to the *WWOX* gene as an example, we observed that the last segment of the genome to be replicated in aphidicolin-treated cells corresponded precisely to the region defined by MiDAS-associated EdU peaks (Fig. 2a, b; Supplementary information, Fig. S2a, b). In the absence of aphidicolin, the same region was again the last one to be replicated, but DNA replication was completed prior to mitotic entry (Fig. 2a, 12 h timepoint after release from the thymidine block; Supplementary information, Fig. S2a).

Genome-wide averages of all the MiDAS regions confirmed the observation that the genomic regions undergoing MiDAS had failed to complete replication prior to mitotic entry (Fig. 2c, d; Supplementary information, Fig. S2c, d). As expected, the average length of the unreplicated regions was significantly larger for the double-peak MiDAS regions than for the single-peak regions. Importantly, in all cases, the MiDAS peaks mapped at the border of the replicated/unreplicated genomic segments. Thus, these results are consistent with MiDAS originating from the collapsed DNA replication forks, where DNA replication stopped upon mitotic entry (Fig. 1d). The double-peak pattern further argues that, at least in these regions, MiDAS does not involve firing of dormant origins within the center of the unreplicated genomic segment.

### DNA replication directionality in MiDAS

**Fig. 3:**
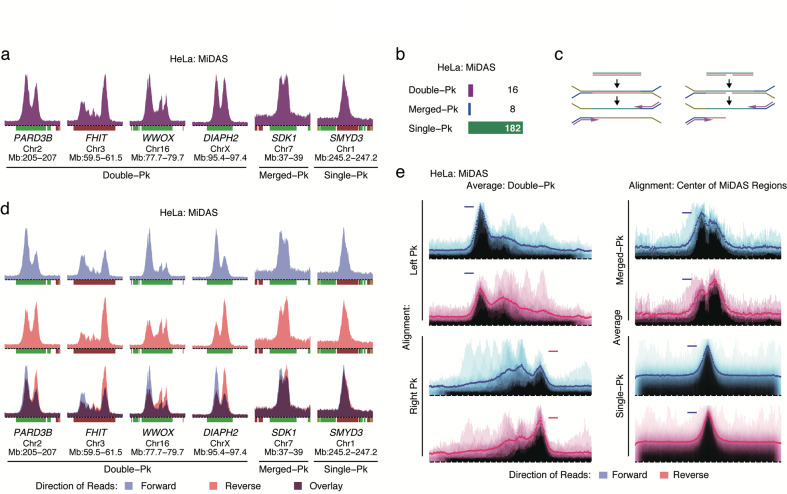
DNA sequencing directionality of MiDAS in HeLa cells. **a** Representative examples of the three types of MiDAS regions in HeLa cells. Peak heights in sigma units: *PARD3B*, 93.8; *FHIT*, 72.7; *WWOX*, 97.0; *DIAPH2*, 162.7; *SDK1*, 39.7; *SMYD3*, 49.4. Bin resolution and ruler scale are as in Fig. 1b. Pk, peak. **b** Number of MiDAS regions of each peak type. **c** Model showing how gaps or nicks in a DNA strand affect library preparation for high throughput sequencing. **d** MiDAS signal at the same genomic locations as in **a**, shown according to sequencing read directionality: forward, blue; reverse, pink; overlay, purple. **e** Genome-wide average MiDAS signal, shown according to sequencing read directionality. The averages were calculated separately for double-peak, merged-peak and single-peak MiDAS regions. The double-peak MiDAS regions were aligned either via their left or right peak, as indicated. The merged- and single-peak MiDAS regions were aligned via their centers. Horizontal lines indicate the maximum average height for each peak type. Span of genomic regions, 2.9 Mb (double-peak) or 2.6 Mb (merged-peak and single-peak); Pk, peak.

To explore the generality of these observations, we examined a second cell line, HeLa, which is derived from a different tissue type than U2OS cells (epithelial, rather than mesenchymal). Using the same protocol as applied for U2OS cells (Fig. 1a; Supplementary information, Fig. S3a), we again observed MiDAS regions that could be classified as double-peak (*n* = 16), merged-peak (*n* = 8) and single-peak (*n* = 182) (Fig. 3a, b). Nascent DNA sequencing in late S phase cells, confirmed that the MiDAS regions corresponded to the genomic domains that had not completed replication prior to mitotic entry (Supplementary information, Fig. S3b, c).

In addition to the standard analysis of the sequencing data, we also examined the directionality of the sequencing reads. High throughput sequencing of double-stranded DNA fragments should reveal no directionality, as either end of the DNA fragment can initiate first-strand synthesis (Fig. 3c, left panel). However, if one of the two DNA strands systematically bears nicks or gaps, then this strand would be underrepresented because it cannot be amplified (Fig. 3c, right panel). Strikingly, the MiDAS reads from HeLa cells revealed sequencing directionality (Fig. 3d). In the double-peak regions, the left peak was overrepresented by forward reads, while the right peak was overrepresented by reverse reads. Genome-wide averages of all the double-peak MiDAS regions confirmed the generality of this observation (Fig. 3e). Directionality was also evident when the merged-peak MiDAS regions were averaged, whereas the averages of the single-peak regions showed no directionality (Fig. 3e), consistent with the hypothesis that the latter regions harbor two peaks that are not resolved from each other (Fig. 1d). A second independent experiment confirmed the directionality of DNA synthesis associated with MiDAS in HeLa cells (Supplementary information, Fig. S3d).

We interpret the observed directionality to indicate that lagging strand synthesis is often delayed compared to leading strand synthesis, since delays in lagging strand synthesis could result in gaps between the Okazaki fragments and an inability to amplify these fragments during library preparation. The directionality is also consistent with the model that MiDAS begins at the ends of the unreplicated genomic regions and proceeds toward the center (Fig. 1d). Interestingly, a similar analysis of the U2OS sequencing data did not reveal directionality, suggesting that U2OS cells couple mitotic leading and lagging strand synthesis more efficiently than HeLa cells (see “Discussion”).

**Fig. 4:**
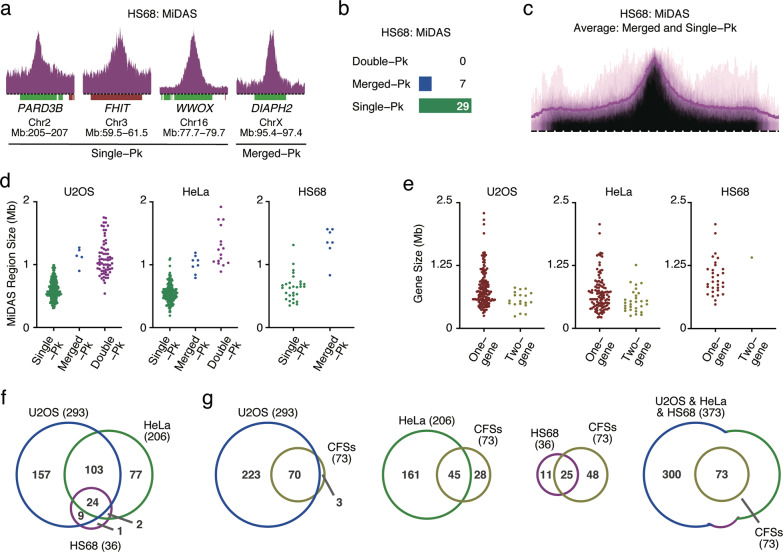
MiDAS regions are shared across cell lines and encompass the known CFSs. **a** Examples of the two types of MiDAS regions present in HS68 cells. Peak heights in sigma units: *PARD3B*, 19.2; *FHIT*, 15.3; *WWOX*, 54.3; *DIAPH2*, 37.8. Bin resolution and ruler scale are as in Fig. 1b. Pk, peak. **b** Number of MiDAS regions of each peak type in HS68 cells. **c** Genome-wide average of MiDAS signal at merged- and single-peak regions in HS68 cells. Span of genomic region, 2.9 Mb. **d** Size distribution of MiDAS regions in U2OS, HeLa and HS68 cells as a function of peak type. **e** Size distribution of genes to which the MiDAS regions map. Gene sizes are plotted separately for the MiDAS regions mapping to one gene or to two adjacent genes. **f**, **g** Venn diagrams showing the overlap between the MiDAS regions identified in the three cell lines (U2OS, blue; HeLa, green; HS68, purple) and CFSs (gold).

Finally, we studied MiDAS in HS68 human fibroblasts, which are not cancer-derived. In these cells, we identified 36 MiDAS regions, which were classified as merged-peak (*n* = 7) and single-peak (*n* = 29) (Fig. 4a–c; Supplementary information, Fig. S4a). No double-peak MiDAS regions were evident in HS68 cells. Thus, the MiDAS regions that were double-peak in U2OS or HeLa cells, were single-peak or even absent in HS68 cells (compare Figs. 3a and 4a). The MiDAS regions that were single-peak in U2OS and HeLa cells were mostly absent in HS68 cells, which explains the much smaller number of MiDAS regions in HS68 cells. We interpret these observations to indicate that HS68 cells generally entered mitosis only after the vast majority of the genome had been replicated. It was not possible to accurately assess directionality of sequencing reads in the HS68 cells because this is best assessed in the context of double-peak MiDAS regions, which were absent in HS68 cells.

### Size and genic composition of MiDAS regions

Next, we set out to characterize the MiDAS regions identified in all three cell lines (Supplementary information, Table S1) with regard to size, genic composition and overlap with CFSs. Plotting the size of the MiDAS regions, as determined by our peak-finding algorithm, revealed that the double-peak MiDAS regions were larger than the merged-peak regions, which, in turn, were larger than the single-peak regions (Fig. 4d). In the three cell lines that we studied, the median sizes of the double-peak regions ranged between 1.1 and 1.2 Mb, whereas the median sizes of the single-peak regions ranged between 0.5 and 0.6 Mb (Fig. 4d).

With regard to genic composition, about two-thirds of the MiDAS regions mapped to one gene; very few mapped to two adjacent genes with a small intergenic sequence between them; and the remaining one-third mapped to intergenic regions (Supplementary information, Fig. S4b). The single genes associated with MiDAS regions were unusually large; in the three cell lines that we studied, their median length ranged between 630 and 930 kb (Fig. 4e). By comparison, the median human gene length is about 55 kb.

### Overlap of MiDAS regions with CFSs and relation to MiDAS foci

The number of MiDAS regions identified by high throughput sequencing ranged from 293 and 206 in U2OS and HeLa cells, respectively, to 36 in HS68 cells. Interestingly, the number of MiDAS foci visualized by microscopy in mitotic cells showed a similar distribution with more foci present in U2OS cells, slightly fewer in HeLa cells and significantly fewer in HS68 cells (Supplementary information, Fig. S4c, d). As mentioned above, adding HU to the media had no impact on the number or intensity of MiDAS foci (Supplementary information, Fig. S4c, d).

At the genome level, the MiDAS regions identified in the three cell lines overlapped to a significant degree. Of the 206 MiDAS regions present in HeLa cells, 127 (62%) were also present in U2OS cells. Moreover, of the 36 MiDAS regions identified in HS68 cells, 35 (97%) were present in U2OS and/or HeLa cells (Fig. 4f).

To monitor the overlap of the MiDAS regions with CFSs, we used a comprehensive list of cytogenetically-defined CFSs.^13^ We focused on 73 CFSs that map to defined genes, since we could assign the position of these CFSs on the genome. A merged list of the MiDAS regions identified in the three cell lines in our study, encompassed all the known 73 CFSs cited above (Fig. 4g). Interestingly, of the 70 MiDAS regions identified in U2OS cells that mapped to known CFSs, 39% were double-peak MiDAS regions. In contrast, of the 223 MiDAS regions identified in U2OS cells that did not map to known CFSs, only 18% were double-peak regions. Thus, the known CFSs correspond preferentially to the larger, double-peak MiDAS regions. Since size is likely to be a factor in identifying CFSs, this may explain why some of the single-peak MiDAS regions have not been identified previously as CFSs.

### CFS-like properties of the MiDAS regions

**Fig. 5:**
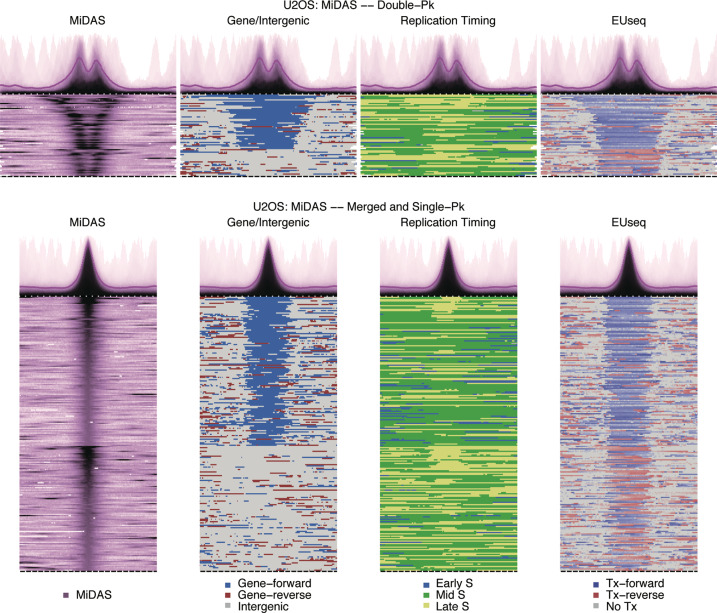
MiDAS regions map to large transcribed units that are mid or late S replicating. Heatmaps showing MiDAS signal, gene annotation, replication timing and transcriptional activity for each MiDAS region and their flanking sequences in U2OS cells. The MiDAS regions mapping to genes are plotted with the gene direction (5′-3′) going from left to right. Replication timing domain data are from untreated U2OS cells. Nascent transcription data are from unsynchronized U2OS cells. Color-coding of the genic/intergenic regions, of the replication timing domains and of the transcribed regions is indicated in the figure. For nascent transcription, the saturation of the blue/red color indicates the level of transcriptional activity at that specific genomic bin. For the double-peak MiDAS regions, the genic regions are plotted first, followed by the intergenic regions; within each subgroup the regions are plotted based on decreasing distance between the two MiDAS peaks. For the merged- and single-peak MiDAS regions, the genic regions are plotted above the intergenic regions and within each subgroup the regions are plotted based on decreasing MiDAS signal. The genome-wide average MiDAS signal is shown above the heatmap plots. Span of genomic regions, 2.9 Mb (double-peak) or 2.3 Mb (merged- and single-peak); Pk, peak, Tx, transcription.

CFSs map to large, late-replicating, transcribed genes,^13–15,19–21^ prompting us to examine the DNA replication and transcription profiles of MiDAS regions. For replication timing, we used our previously-generated, genome-wide data derived from untreated U2OS cells.^53^ For transcriptional activity, we sequenced the nascent transcripts of unsynchronized U2OS, HeLa and HS68 cells. In all three cell lines, the MiDAS regions mapped to either mid S or late S phase replicating genomic domains (Fig. 5; Supplementary information, Fig. S5). In addition, all the MiDAS regions, even those that did not map to genes, corresponded to actively transcribed genomic domains. Importantly, there was a strong concordance between the size of the MiDAS region and the length of the transcribed domain (Fig. 5; Supplementary information, Fig. S5).

To further probe the link between MiDAS and transcriptional activity, we took advantage of the fact that certain genomic regions exhibited MiDAS in U2OS, but not in HeLa cells (Fig. 4f). These regions were transcriptionally active in U2OS cells, but not in HeLa cells (Supplementary information, Fig. S6a), providing a very strong correlation between MiDAS and transcriptional activity.

We also focused on the intergenic genomic regions that exhibited MiDAS in U2OS cells. All these regions were transcriptionally active in U2OS cells, but in HS68 cells most of these regions were transcriptionally inactive and did not exhibit MiDAS (Supplementary information, Fig. S6b). These data are consistent with there being a causative role for transcriptional activity in triggering MiDAS and also revealed that transcriptional activity in intergenic regions is more prevalent in cancer cells, than in normal cells, possibly linked to cancer-associated genomic rearrangements and/or epigenetic changes.

### Paucity of replication origin firing within MiDAS regions

**Fig. 6:**
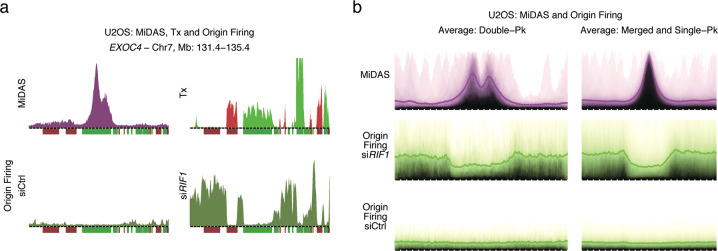
MiDAS regions are mostly devoid of DNA replication origins. **a** Top panels, MiDAS signal (purple) and nascent transcription (green, forward transcription; red, reverse transcription) of non-transfected U2OS cells. Bottom panels, origin firing profiles (olive-green) of control (Ctrl) and RIF1-depleted U2OS cells at the *EXOC4* locus. For origin firing, the cells were synchronized by mitotic shake-off, released from the mitotic block and allowed to progress through G1 and into S phase over 14 h in the presence of EdU, to label nascent DNA, and hydroxyurea, to limit fork progression. Under these conditions, in control cells, origin firing is observed only in the early S-replicating part of the genome. Bin resolution and ruler scale are as in Fig. 1b. Tx, transcription. **b** Genome-wide average origin firing signal (green) in control and RIF1-depleted cells at double-peak (left panels) and merged- and single-peak (right panels) MiDAS regions, in comparison to the genome-wide average MiDAS signal (purple). Span of genomic region, 2.9 Mb (double-peak) or 2.3 Mb (merged- and single-peak); Pk, peak.

Previous analysis of a very small number of CFSs has suggested that few if any DNA replication origins fire within CFSs.^22,24^ We examined whether the same applied for MiDAS regions. For this analysis, we were unable to use our recently described method to monitor origin firing,^52^ since this method can only interrogate firing of early S origins, whereas the MiDAS regions map to mid S- and late S-replicating genomic domains. In both lower and higher eukaryotes, inactivation of the RIF1 protein deregulates the timing of origin firing, such that mid S and late S origins fire prematurely.^54–56^ Hence, we depleted RIF1 in U2OS cells (Supplementary information, Fig. S7a) and monitored origin firing genome-wide. As reported previously, RIF1 depletion induced premature origin firing in mid S- and late S-replicating domains (Fig. 6a, b; Supplementary information, Fig. S7b). However, origin firing was generally lacking within MiDAS regions, as shown by the example of the *EXOC4* locus and, also, genome-wide (Fig. 6a, b). Reduced firing of replication origins within MiDAS regions means that their replication would be dependent on flanking origins; hence, DNA replication forks would have to travel very long distances to complete replication. We propose that this can help explain why the MiDAS regions are not fully replicated prior to mitotic entry in cells treated with aphidicolin.

### Mapping of cancer genome rearrangement breakpoints to MiDAS regions

CFSs are frequently the sites of chromosomal rearrangements in cancer,^9,12,19,29,57–62^ which prompted us to examine whether cancer genome rearrangement breakpoints map to MiDAS regions. We interrogated a dataset of approximately half a million genomic rearrangements identified in five thousand cancers across twelve different cancer types.^61^ From this dataset, we retained only the simple genomic deletions and amplifications, for which both breakpoint termini mapped within 1 Mb of the center of a MiDAS region. This allowed us to examine the focal genomic rearrangements that mapped within MiDAS regions.

We performed this analysis using the list of MiDAS regions identified in U2OS cells. The MiDAS regions were split into four groups: mid S-replicating double-peak regions (*n* = 33); late S-replicating double-peak regions (*n* = 34); mid S-replicating merged-peak and single-peak regions (*n* = 123); and late S-replicating merged-peak and single-peak regions (*n* = 103). The replication timing in the above classification refers to the replication timing at the center of the MiDAS region.

**Fig. 7:**
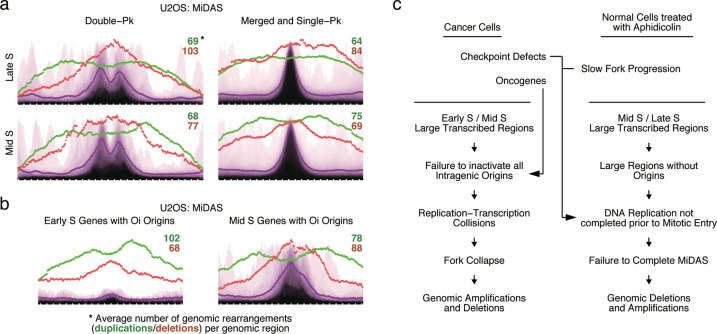
Mapping of human cancer genomic rearrangements to MiDAS regions and model for induction of genomic instability at CFSs. **a** Mapping of human cancer focal duplications and deletions at the MiDAS regions identified in U2OS cells. The MiDAS regions were classified as mid S or late S, according to the replication timing of the genomic bin at their center. For each genomic bin, the number of genomic rearrangements spanning that genomic bin is plotted (deletions, red; duplications, green). The average number of focal duplications and deletions per MiDAS region is indicated at the top right of each graph. The genome-wide average MiDAS signal is shown in purple. Span of genomic region, 2.9 Mb (double-peak) or 2.3 Mb (merged- and single-peak); Pk, peak. **b** Mapping of human cancer focal duplications and deletions at early and mid S-replicating, large, transcribed genes, previously shown to harbor oncogene-induced (Oi) DNA replication origins. The genomic rearrangements and genome-wide average MiDAS signal are plotted as in **a**. Span of genomic region, 2.3 Mb. **c** Proposed model to explain the origin of genomic rearrangements in cancer cells and in normal cells treated with aphidicolin.

For each of the above groups, we plotted the number of deletions and duplications that mapped to each genomic bin. The number of deletions peaked at the center of the MiDAS regions (Fig. 7a), which is consistent with deletions arising from an inability to complete replication of these genomic domains. In contrast, the number of duplications showed two peaks that flanked the MiDAS regions (Fig. 7a). We speculate that duplications arise when a converging fork replicates beyond the site of a collapsed fork; in this case, a genomic segment would be replicated twice: once by the converging fork and once by the fork initiated by BIR repair of the collapsed fork (Supplementary information, Fig. S8).^41^ Thus, duplications may not indicate a failure to complete replication, but rather an error in repair of collapsed DNA replication forks. Consistent with this interpretation, deletions were more frequent than duplications in the late S-replicating, double-peak MiDAS regions, since in this case large genomic segments need to be replicated within a short time window in late S phase. In contrast, duplications were more frequent than deletions in the mid S-replicating, merged-peak and single-peak regions, in which case there is a larger time window to replicate a relatively shorter genomic segment (Fig. 7a).

Since the formation of duplications cannot easily be explained by a failure to complete DNA replication, we infer that some genomic rearrangements targeting MiDAS regions and CFSs in cancer are likely due to replication-transcription conflicts, rather than being simply due to delayed, but unperturbed, replication of the locus. Indeed, oncogenes induce the firing of ectopic DNA replication origins within genes and, particularly, within large genes. We had previously identified sets of large genes that harbor oncogene-induced DNA replication origins.^53^ These genes map to early (*n* = 26) or mid (*n* = 28) S-replicating genomic domains. We plotted the human cancer genomic rearrangements that map to these genes and found that duplications were more frequent than deletions in the early S genes, whereas the inverse was true for the mid S genes (Fig. 7b). Not unexpectedly, 25 of the 28 mid S-replicating large genes harboring oncogene-induced DNA replication origins also exhibited MiDAS, since large, transcriptionally active genomic domains favor both firing of oncogene-induced origins and MiDAS (Fig. 7c).

## Discussion

### MiDAS permits CFSs to be mapped at high resolution on the human genome

Previously, the study of MiDAS was limited to inspection of EdU-positive foci on mitotic chromosomes.^37,44^ Using a variant of EdUseq,^52^ we mapped the genomic coordinates of MiDAS at high resolution. Depending on the cells studied, the number of MiDAS regions ranged between 36 and 293. Merging of all the MiDAS regions across the three cell lines, led to the identification of a total of 373 unique loci undergoing MiDAS. These loci included all the 73 human CFSs previously mapped to large genes.^13^ The remaining MiDAS regions also have features characteristic of CFSs, as they corresponded to large, transcribed, origin-poor, mid S- or late S-replicating genomic domains. The larger number of MiDAS regions identified in our study compared to the known CFSs most likely reflects the much higher sensitivity of the EdUseq method, as compared to observing chromosomal breaks or gaps on mitotic chromosomes. We suggest, therefore, that the list of MiDAS regions identified here provides a more definitive and comprehensive resource of CFSs than has been available hitherto.

### MiDAS appears to be a BIR repair pathway for completing duplication of the genome

Previous studies had established the presence of MiDAS in cells treated with aphidicolin, but had not determined the physiological role of such DNA synthesis.^37,44^ Our results strongly support the hypothesis that MiDAS serves to complete genome duplication in cells that enter mitosis without having fully replicated their genomic DNA. Monitoring DNA synthesis genome-wide in late S phase cells treated with aphidicolin revealed genomic regions that failed to be replicated even 24 h after the cells had entered S phase. These regions corresponded precisely to the genomic regions that exhibited MiDAS upon mitotic entry.

MiDAS is dependent on *POLD3* and other genes that function in BIR.^37,43–48^ Accordingly, it has been proposed that MiDAS is a form of BIR.^44^ Two observations from our current study support this premise. First, the MiDAS peaks map to the boundaries of the replicated/unreplicated genomic regions in late S cells. This pattern is consistent with the ongoing DNA replication forks collapsing when cells enter mitosis, and then DNA replication re-initiating from the sites of the collapsed forks through BIR. Indeed, high levels of mitotic cyclin-dependent kinases induce fork collapse, as shown in various experimental systems.^63^ The alternative scenario of MiDAS being driven by the firing of dormant origins within the body of the unreplicated genomic regions would have resulted in DNA replication being more prominent at the center of the unreplicated segments, rather than at their borders. The sequence directionality of the MiDAS-driven EdU incorporation in HeLa cells further supports the notion that MiDAS is a form of BIR. Although such sequencing directionality has not, to our knowledge, been described previously, it is consistent with BIR, where leading and lagging strand synthesis can be dissociated.^49,50^

We did not observe sequencing directionality in HS68 and U2OS cells. However, in HS68 cells, the MiDAS regions were single-peak, making it difficult to observe sequencing directionality. U2OS cells utilize the alternative-lengthening of telomeres (ALT) pathway to maintain telomere length. Since ALT is mediated by BIR,^51,64,65^ it is possible that U2OS cells have become highly proficient in BIR, thereby minimizing the temporal dissociation of leading and lagging strand synthesis.

### Mechanisms for induction of genomic instability at CFSs

A number of mechanisms have been proposed to explain the presence of genomic instability at CFSs.^9,11,26–29^ One model posits that CFSs contain regions that are unusually hard to replicate due to their sequence composition, thereby explaining why these regions remain unreplicated in cells entering mitosis.^36^ Our analysis is largely inconsistent with this model, as the position of the MiDAS peaks in cells entering mitosis varied depending on the concentration of aphidicolin used. Nevertheless, it remains possible that sequences that are prone to secondary structure formation impede MiDAS rather than conventional DNA replication, thereby resulting in chromosomal breaks and/or gaps.

A second model posits that the genomic organization of CFSs is such that these regions require a very long time to replicate.^9,10,22,24^ Our findings support this model. The MiDAS regions, which encompass the known CFSs, map to large, late-replicating, origin-poor genomic domains that are, therefore, at risk of failing to complete DNA replication before cells enter mitosis.^9,10^ Indeed, replication of the MiDAS regions is mediated by two converging DNA replication forks, each of which needs to travel distances of 200–700 kb.

The reason MiDAS regions lack DNA replication origins can be attributed to the presence of transcriptional activity. Transcription suppresses origin firing^53^ and the MiDAS regions are universally transcriptionally active. Thus, suppression of replication origin firing over large, late-replicating genomic regions by transcription, could result in an inability to complete replication of these regions prior to mitotic entry and, hence, to genomic instability. This sequence of events explains why exposure of cells to aphidicolin enhances dramatically the presence of genomic instability at CFSs, since aphidicolin slows down fork progression and makes it even less likely that CFSs will be replicated prior to cell division.

### Relevance to cancer

Recently, we described a mechanism to explain how oncogenes induce genomic instability in cancer.^53^ Briefly, we observed that oncogenes induce firing of DNA replication origins within transcribed genes, resulting in replication-transcription conflicts, formation of DNA double-strand breaks and genomic instability. In normal cells, intragenic replication origins are inactivated by transcription during the G1 phase of the cell cycle. However, in cancer cells, the oncogene-induced shortening of the length of the G1 phase leaves less time for transcription to inactivate all intragenic origins, particularly those origins that are present within very large genes.

The genomic features associated with oncogene-induced DNA replication origins and with MiDAS are similar; very large, transcribed genes. Accordingly, we view genomic instability at CFSs in cancer as being due to two mechanisms: (i) replication-transcription conflicts due to the firing of oncogene-induced origins and (ii) failure to complete DNA replication prior to mitotic entry (Fig. 7c). The first mechanism would lead to fork collapse and repair by BIR, which in turn could lead to genomic duplications at the borders of the CFSs (Supplementary information, Fig. S8). The second mechanism would lead to genomic deletions at the central portions of the CFSs. Oncogene-induced DNA replication origins fire predominantly in the early S- and mid S-replicating genomic domains, whereas failure to complete DNA replication prior to mitotic entry is more relevant in the context of mid S- and late S-replicating domains. Accordingly, in human cancers, focal genomic duplications are more prevalent in mid S-replicating CFSs, whereas focal genomic deletions are more prevalent in late S-replicating CFSs (Fig. 7a).

Given the high frequency of genomic instability at CFSs in cancer, the mapping of MiDAS regions throughout the genome could help identify cancers that are experiencing DNA replication stress. There is clearly a need for the development of a good biomarker for DNA replication stress in the clinic, particularly considering that several drugs that target DNA replication stress, including ATR and Chk1 inhibitors, are currently being evaluated in clinical trials.^66–68^ Thus, the precise mapping of MiDAS regions, reported here and in an accompanying paper,^69^ will accelerate the development of reliable clinical biomarkers.

## Materials and methods

### Cell culture

U2OS, HeLa and HS68 cells were maintained at 37 °C in Dulbecco’s modified Eagle’s medium (Invitrogen, Cat. No. 11960) supplemented with penicillin-streptomycin-glutamine (Invitrogen, Cat. No. 10378-016) and either 10% fetal bovine serum (FBS; Invitrogen, Cat. No. 10500) for U2OS and HeLa cells, or 15% fetal bovine serum for HS68 cells. For some experiments, a clone of U2OS cells that can inducibly overexpress cyclin E (U2OS-CE) was used. For this clone, the culture medium was additionally supplemented with 400 μg/mL G418 (Invitrogen, Cat. No. 10131-027), 1 μg/mL puromycin (Sigma, Cat. No. P8833) and 2 μg/mL tetracycline (Sigma, Cat. No. T7660). Under these conditions, ectopic cyclin E protein levels are maintained at low levels and the cells behave as normal U2OS cells.^41,45,57^

### Antibodies used for immunoblotting

Immunoblotting were performed using the following antibodies: RIF1 (Bethyl, Cat. No. A300-569A) and α-Actinin (Millipore, Cat. No. 05–384).

### MiDAS

#### Main protocol

To synchronize the cells at the G1/S transition, asynchronously growing cell cultures, at 70% confluency, were treated with 2 mM thymidine (Sigma-Aldrich, T1895). The thymidine block was maintained for 18 h in U2OS and HeLa cells and for 30 h in HS68 cells. The cells were then washed four times with warm PBS and were released into warm medium containing aphidicolin (0.4 µM, unless specifically indicated otherwise) (Sigma-Aldrich, A0781) and RO3306 (9 µM) (APExBio, A8885), to respectively expose the cells to replication stress and arrest them at the G2/M boundary. U2OS and HeLa cells were both released for 16 h, whereas HS68 cells were released for 30 h, as they needed a longer release period to complete S phase under these conditions. The G2-arrested cells were then thoroughly washed three times with warm medium for 5 min and were released for 1 h into warm medium containing 200 ng/mL nocodazole (Tocris, Cat. No. 1228) and 10 µM EdU (Thermo Fisher, A10044) to label DNA synthesis in early mitosis and subsequently arrest them in prometaphase. The nocodazole-arrested cells were then collected by mitotic shake-off and fixed with ice-cold 90% methanol overnight at −20 °C. Because a small proportion of the cells had not yet reached G2 phase at the time of the EdU treatment, 2 mM hydroxyurea (Sigma-Aldrich, H8627) was also added to the cells for the last 1.5 h of the RO3306 treatment and for the entire time of the EdU and nocodazole treatment, to suppress the EdU signal from any S phase cells that could potentially detach during the mitotic shake-off. Biotinylation and isolation of EdU-labeled DNA was then performed as described previously.^52,53^ Briefly, after fixation, the cells were permeabilized with 0.2% Triton-X in PBS, and EdU was attached to a cleavable biotin linker (Azide-PEG(3 + 3)-S-S-biotin) (Jena Biosciences, Cat. No. CLK-A2112-10) using the reagents of the Click-it Kit (Invitrogen, Cat. No. C-10424). The DNA was isolated by phenol-chloroform extraction and ethanol precipitation and then sonicated to a size of 100–500 bp. Dynabeads MyOne streptavidin C1 (Invitrogen, Cat. No. 65001) were used to isolate the EdU-labeled DNA fragments. The beads were washed three times with Binding and Washing Buffer 1× (5 mM Tris-HCl, pH 7.5, 0.5 mM EDTA, 1 M NaCl, 0.5% Tween-20) and then resuspended in twice their original volume with an equal volume of Binding and Washing Buffer 2× containing the sonicated DNA. The DNA-bead mixture was incubated 15 min at room temperature with rotation, prior to washing the beads three times with Binding and Washing Buffer 1× and once with TE buffer (10 mM Tris-HCl, pH 8.0, 10 mM EDTA). To elute the EdU-labeled DNA from the streptavidin beads, 2% β-mercaptoethanol (Sigma, Cat. No. M6250) was added for 1 h at room temperature. The eluted DNA was finally used for library preparation. Sequencing libraries were made by the Genomics Platform of the University of Geneva using the TruSeq ChIP Sample Prep Kit (Illumina, Cat. No. IP-202-1012). One hundred base pair single-or paired-end read sequencing reactions were then performed on an Illumina Hi-Seq 4000 sequencer.

#### Alternate protocol

For the alternate MiDAS protocol, a different synchronization strategy was used in order to study DNA synthesis in cells that had already entered mitosis. Asynchronously growing cells, at 70% confluency, were treated for 8 h with 100 ng/mL nocodazole (Tocris, Cat. No. 1228) and 0.4 µM aphidicolin (Sigma-Aldrich, A0781) to induce DNA replication stress in S phase cells before arresting them in prometaphase. Negative control cells were obtained by treating the cells with nocodazole but omitting aphidicolin. After the 8 h treatment, mitotic cells were shaken-off, washed with PBS and released for 4 h into warm medium with 2 mM hydroxyurea (Sigma, Cat. No. H8627) and 25 µM EdU (Invitrogen, Cat. No. A10044) to label DNA synthesis from prometaphase to early G1 phase. The cells were finally collected by trypsinization, fixed with 90% methanol overnight and EdU-labeled DNA was isolated and sequenced as described in the main MiDAS protocol.

### Late S phase DNA synthesis

To map the genomic regions that replicate in late S phase, asynchronously growing cells, at 70% confluency, were treated with 2 mM thymidine (Sigma-Aldrich, T1895) for 18 h, then washed four times with warm PBS and released for various time periods in warm medium with or without low doses aphidicolin (0.4 µM) (Sigma-Aldrich, A0781) and with or without RO3306 (9 µM) (APExBio, A8885). EdU (25 µM) (Invitrogen, Cat. No. A10044) was added to the medium 30 min prior to harvesting and fixing the cells. The EdU-labeled DNA was then isolated and sequenced as described in the main MiDAS protocol.

### EUseq

EU (5-Ethynyl-uridine; Jena Biosciences, Cat. No. CLK-N002-10) was added to asynchronously growing U2OS, HeLa and HS68 cells for 30 min. The cells were then collected and RNA was extracted and purified using TRIzol (Invitrogen, Cat. No. 15596) and isopropanol precipitation. EU-labeled RNA was attached to a cleavable biotin-azide linker (Azide-PEG(3 + 3)-S-S-biotin) (Jena Biosciences, Cat. No. CLK-A2112-10) using the reagents of the Click-iT Nascent RNA Capture Kit (Invitrogen, Cat. No. C-10365) according to the manufacturer’s instructions. The purified RNA was heated at 70 °C and placed on ice to remove secondary structures. EU-labeled RNA was then isolated using Dynabeads MyOne streptavidin C1 (Invitrogen, Cat. No. 65001) as described previously.^53^ The streptavidin beads were washed three times with Binding and Washing Buffer 1× (5 mM Tris-HCl, pH 7.5, 0.5 mM EDTA, 1 M NaCl, 0.5% Tween-20) followed by two 2 min washes in Solution A (0.1 M NaOH, 0.05 M NaCl) and two washes in Solution B (0.1 M NaCl). The beads were resuspended in twice their original volume with an equal volume of Binding and Washing Buffer 2× and the RNA. The mix was incubated for 30 min at room temperature with rotation and the beads were washed three times with Binding and Washing Buffer 1× and once with RNAse-free-water. The EU-labeled RNA was finally eluted by incubating the streptavidin beads with 2% β-mercaptoethanol (Sigma, Cat. No. M6250) for 1 h at room temperature. Sequencing libraries were made by the Genomics Platform of the University of Geneva using the TruSeq Stranded Total RNA with Ribo-Zero Gold (Illumina, Cat. No. RS-122-2301) omitting the ribo-depletion step. One hundred base pair single-end read sequencing reactions were performed on an Illumina Hi-Seq 4000 sequencer.

### EdUseq in RIF1-depleted-cells

U2OS-CE with normal levels of cyclin E were transfected with siRNAs targeting *RIF1* (AAGAAUGAGCCCCUAGGGAAAdTdT)^70^ using the Amaxa^®^ Cell Line Nucleofector^®^ Kit V (Lonza) according to the manufacturer’s instructions. Two days later, nocodazole (Tocris, Cat. No. 1228) was added to the cells at a concentration of 100 ng/mL for 8 h. Mitotic cells were shaken-off, washed with PBS and released into warm medium with 2 mM hydroxyurea (Sigma, Cat. No. H8627) and 25 µM EdU (Invitrogen Cat. No. A10044). The cells were collected 14 h later and fixed with 90% methanol overnight. EdU-labeled DNA was attached to a cleavable biotin linker (Azide-PEG(3 + 3)-S-S-biotin) (Jena Biosciences, Cat. No. CLK-A2112-10), isolated and subjected to high-throughput sequencing as described above. Sequencing libraries were made by the Genomics Platform of the University of Geneva using the TruSeq ChIP Sample Prep Kit (Illumina, Cat. No. IP-202-1012). One hundred base pair single-end read sequencing reactions were then performed on an Illumina Hi-Seq 2500 sequencer.

### Sequencing read alignment and processing

Sequencing reads were aligned and processed as described in detail previously.^52^ Briefly, the Burrows-Wheeler Aligner software was used to align the reads on the masked human genome assembly (GRCh37/hg19) retaining only the reads with the highest quality score. Custom Perl scripts, all provided previously,^52^ were then used to assign the aligned reads to 10 kb genomic bins; the data were normalized, sigma values were calculated and the data visualized, as described previously.^52^

### Identification and classification of MiDAS peaks

A custom Perl script was used to identify genomic regions with sigma values that were significantly higher than the sigma values of the background. Peak searching within these regions was then performed to identify the positions of the peaks and the number of peaks present in each genomic region. The genomic regions with MiDAS signal were then classified as double-peak, merged-peak and single-peak, depending on the number of peaks present in each genomic region.

The peaks were plotted using sigma values, which takes into account systemic biases in high throughput sequencing data.^52,53^ These biases relate to some genomic regions being better substrates for NGS library preparation and to differences in the extent to which highly repetitive sequences are present in various genomic bins. To correct for these biases, for each genomic bin we divided the number of EdUseq/EUseq sequence reads by the number of reference reads for the same genomic bin (the reference was calculated from sequencing of genomic DNA from the same cell line, performed at very high coverage). This gave the adjusted number of EdUseq/EUseq reads. We then calculated the standard deviation associated with each genomic bin. The sigma value is the adjusted number of EdUseq/EUseq reads divided by its standard deviation. For plots of individual genomic regions, we presented the peak sigma values. For the plots showing averages of MiDAS peaks, we adjusted the height of the peaks, so that each MiDAS peak contributed equally to the plot (to avoid a small number of very strong peaks dominating the average). Because each peak was scaled by a different factor, it is not valid to calculate sigma values for the peak averages.

## Supplementary information

Supplementary Figure S1

Supplementary Figure S2

Supplementary Figure S3

Supplementary Figure S4

Supplementary Figure S5

Supplementary Figure S6

Supplementary Figure S7

Supplementary Figure S8

Supplementary Table S1

## Data Availability

The fastq sequencing data and associated information described in this study have been deposited in the Sequence Read Archive (SRA) as BioProject PRJNA588267.

## Code availability

Computer codes and data files used to process and plot the data are available from our previous publications.^52,53^ Peak-finding scripts are available upon request.
