# Supplementary material for: High-resolution mapping of mitotic DNA synthesis regions and common fragile sites in the human genome through direct sequencing
Source: Cell Res. 2020 Jun 19;30(11):997–1008. doi: 10.1038/s41422-020-0358-x (PMC7784693; doi:10.1038/s41422-020-0358-x)
Supplement: Supplementary file 1 — Supplementary Figure S1 [file 41422_2020_358_MOESM1_ESM.pdf]

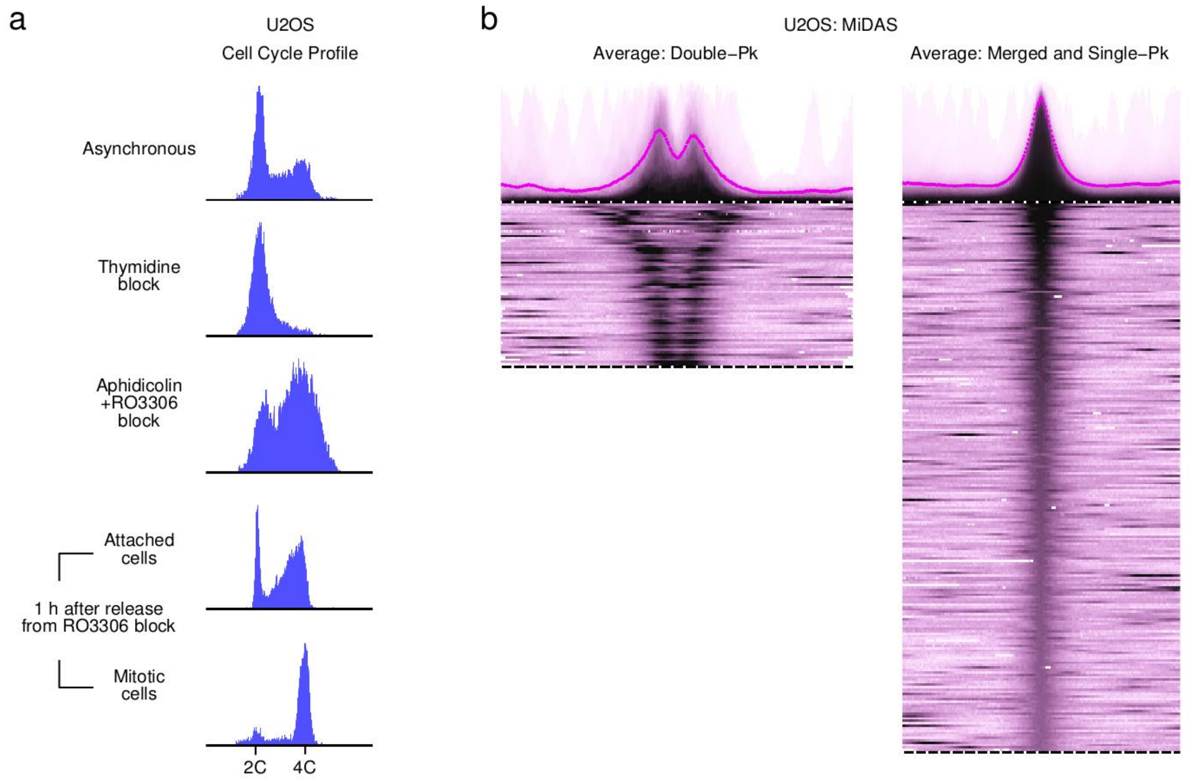

Supplementary Fig. S1. Cell cycle profiles and MiDAS heatmaps of U2OS cells
a Cell cycle profile of asynchronous and synchronized U2OS cells, as determined by flow cytometry after staining of the genomic DNA with propidium iodide. The cells were treated according to the protocol shown in Fig. 1a.
b Heatmaps showing the MiDAS signal for each MiDAS region and their flanking sequences in U2OS cells. The genome-wide average MiDAS signal for each peak type is shown above the heatmaps. Span of genomic region, 2.9 Mb (double-peak) or 2.3 Mb (merged and single-peak); Pk, peak.
