# Supplementary material for: High-resolution mapping of mitotic DNA synthesis regions and common fragile sites in the human genome through direct sequencing
Source: Cell Res. 2020 Jun 19;30(11):997–1008. doi: 10.1038/s41422-020-0358-x (PMC7784693; doi:10.1038/s41422-020-0358-x)
Supplement: Supplementary file 2 — Supplementary Figure S2 [file 41422_2020_358_MOESM2_ESM.pdf]

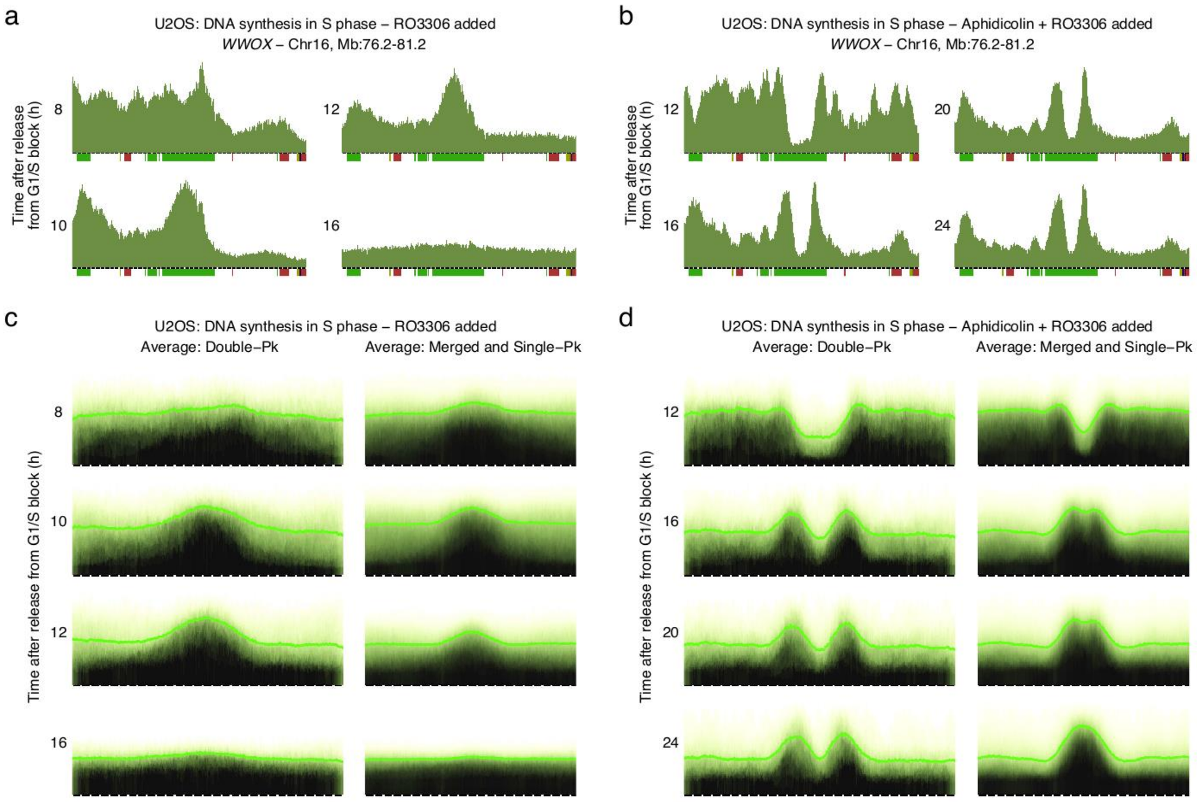

Supplementary Fig. S2. Late S replication profiles of U2OS cells treated with RO3306 and optionally with aphidicolin

a-b Replication signal (green) around the *WWOX* gene at the indicated times after release from a thymidine block, in the presence of RO3306 (**a**) or RO3306 and aphidicolin (**b**). Bin resolution and ruler scale are as in Fig. 1b.

c-d Genome-wide average replication signal at double-peak (left panels; span of genomic region, 2.9 Mb) and merged- and single-peak (right panels; span of genomic region, 2.3 Mb) MiDAS regions at the indicated times after release from a thymidine block, in the presence of RO3306 (**c**) or RO3306 and aphidicolin (**d**). Pk, peak.
