# Supplementary material for: High-resolution mapping of mitotic DNA synthesis regions and common fragile sites in the human genome through direct sequencing
Source: Cell Res. 2020 Jun 19;30(11):997–1008. doi: 10.1038/s41422-020-0358-x (PMC7784693; doi:10.1038/s41422-020-0358-x)
Supplement: Supplementary file 3 — Supplementary Figure S3 [file 41422_2020_358_MOESM3_ESM.pdf]

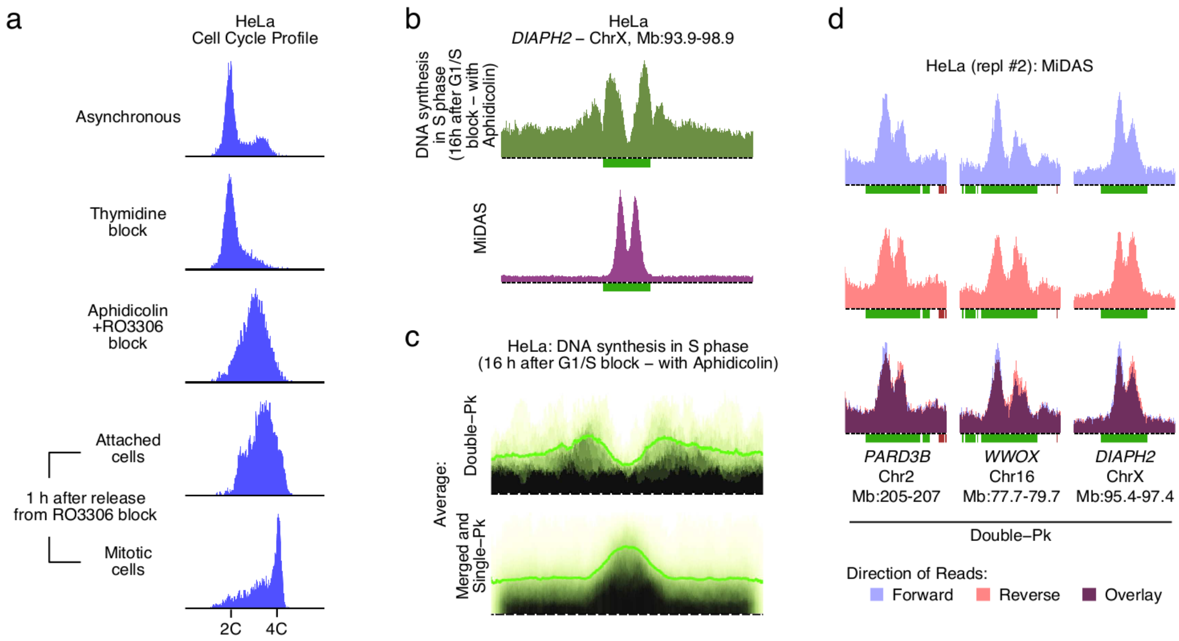

Supplementary Fig. S3. Cell cycle profiles, late S replication profiles and MiDAS DNA sequencing directionality in HeLa cells

a Cell cycle profile of asynchronous and synchronized HeLa cells, as determined by flow cytometry after staining of the genomic DNA with propidium iodide. The cells were treated according to the protocol shown in Fig. 1a.

b Replication signal (green) around the *DIAPH2* gene 16 h after release from a thymidine block in HeLa cells treated with aphidicolin (0.4 µM), compared to the MiDAS signal (purple). Bin resolution and ruler scale are as in Fig. 1b.

c Genome-wide average replication signal at double-peak and merged- and single-peak MiDAS regions, 16 h after release from a thymidine block in HeLa cells treated with aphidicolin (0.4 µM). Span of genomic region, 2.7 Mb.

d MiDAS signal at three genomic sites, shown according to sequencing read orientation: forward (blue), reverse (pink) and overlay (purple). The data are derived from a second replicate (repl) of the experiment shown in Fig. 3d.
