# Supplementary material for: High-resolution mapping of mitotic DNA synthesis regions and common fragile sites in the human genome through direct sequencing
Source: Cell Res. 2020 Jun 19;30(11):997–1008. doi: 10.1038/s41422-020-0358-x (PMC7784693; doi:10.1038/s41422-020-0358-x)
Supplement: Supplementary file 4 — Supplementary Figure S4 [file 41422_2020_358_MOESM4_ESM.pdf]

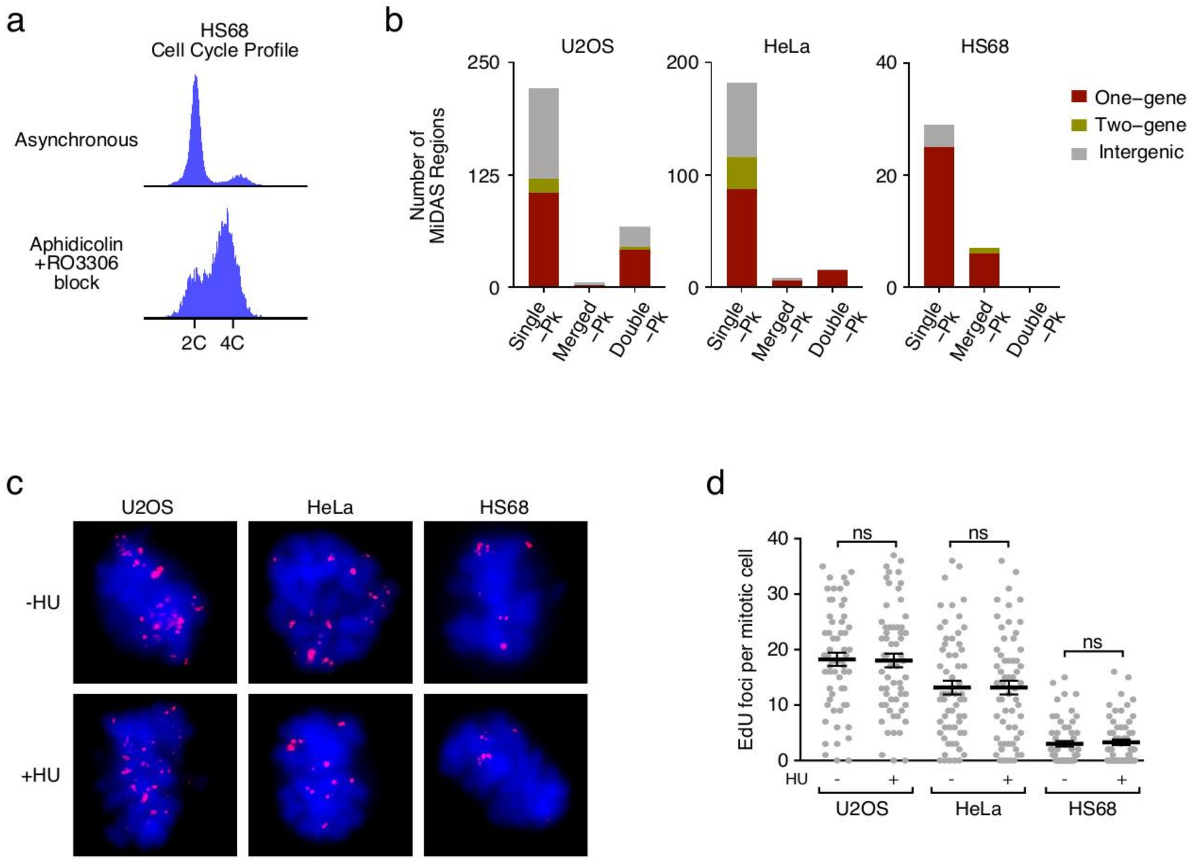

Supplementary Fig. S4. Cell cycle profiles of HS68 cells and mapping of MiDAS regions to genic and intergenic regions

a Cell cycle profile of asynchronous and synchronized HS68 cells, as determined by flow cytometry after staining of the genomic DNA with propidium iodide. The cells were treated according to the protocol shown in Fig. 1a.

b Bar plot showing the number of MiDAS regions mapping to one gene (One-gene, red), two adjacent genes (Two-gene, yellow) or to intergenic sequences (Intergenic, grey). Pk, peak.

c-d EdU foci in cells treated with the protocol in Fig. 1a with and without adding hydroxyurea (HU) to the medium. The cells were visualized by microscopy (**c**) and the number of EdU foci per mitotic cell was counted (**d**; N=60 cells per condition). The bars indicate the mean +/- one standard error of the mean. ns, not statistically significant.
