# Supplementary material for: High-resolution mapping of mitotic DNA synthesis regions and common fragile sites in the human genome through direct sequencing
Source: Cell Res. 2020 Jun 19;30(11):997–1008. doi: 10.1038/s41422-020-0358-x (PMC7784693; doi:10.1038/s41422-020-0358-x)
Supplement: Supplementary file 5 — Supplementary Figure S5 [file 41422_2020_358_MOESM5_ESM.pdf]

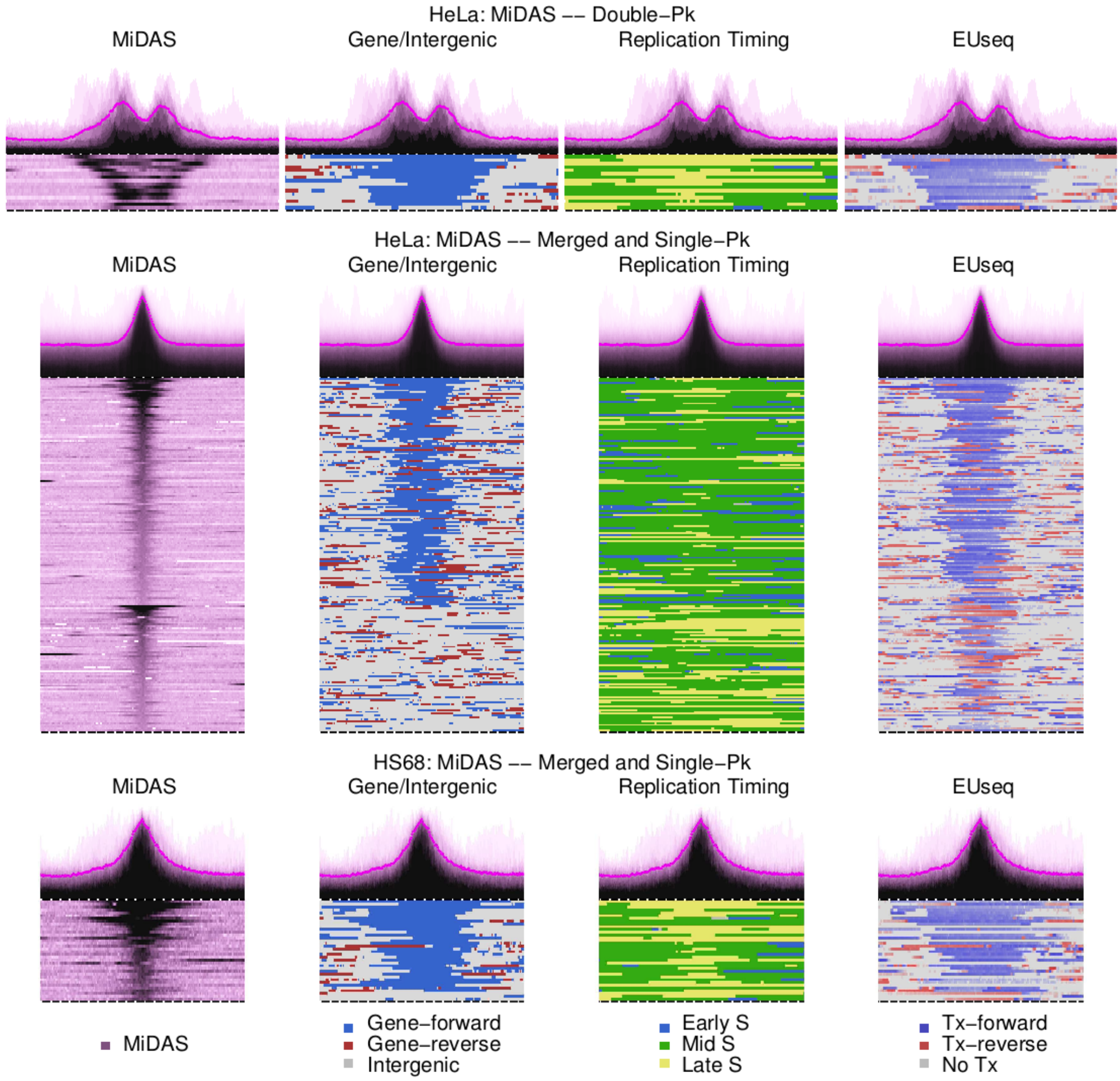

Supplementary Fig. S5. MiDAS regions in HeLa and HS68 cells map to large transcribed units that are mid or late S replicating

Heatmaps showing MiDAS signal, gene annotation, replication timing and transcriptional activity for each MiDAS region and their flanking sequences in HeLa and HS68 cells. The MiDAS regions mapping to genes are plotted with the gene direction (5'-3') going from left to right. Replication timing domain data are from untreated U2OS cells. Nascent transcription data are from unsynchronized HeLa and HS68 cells. Color-coding of the genic/intergenic regions, of the replication timing domains and of the transcribed regions is indicated in the figure. For nascent transcription, the saturation of the blue/red color indicates the level of transcriptional activity at that specific genomic bin. For the double-peak MiDAS regions, the genic regions are plotted first, followed by the intergenic regions; within each subgroup the regions are plotted based on decreasing distance between the two MiDAS peaks. For the merged and single-peak MIDAS regions, the genic regions are plotted above the intergenic regions and within each subgroup the regions are plotted based on decreasing MIDAS signal. The genome-wide average MiDAS signal is shown above the heatmap plots. Span of genomic regions, 2.9 Mb (double-peak) or 2.3 Mb (merged and single-peak); Pk, peak; Tx, transcription.
