# Supplementary material for: High-resolution mapping of mitotic DNA synthesis regions and common fragile sites in the human genome through direct sequencing
Source: Cell Res. 2020 Jun 19;30(11):997–1008. doi: 10.1038/s41422-020-0358-x (PMC7784693; doi:10.1038/s41422-020-0358-x)
Supplement: Supplementary file 6 — Supplementary Figure S6 [file 41422_2020_358_MOESM6_ESM.pdf]

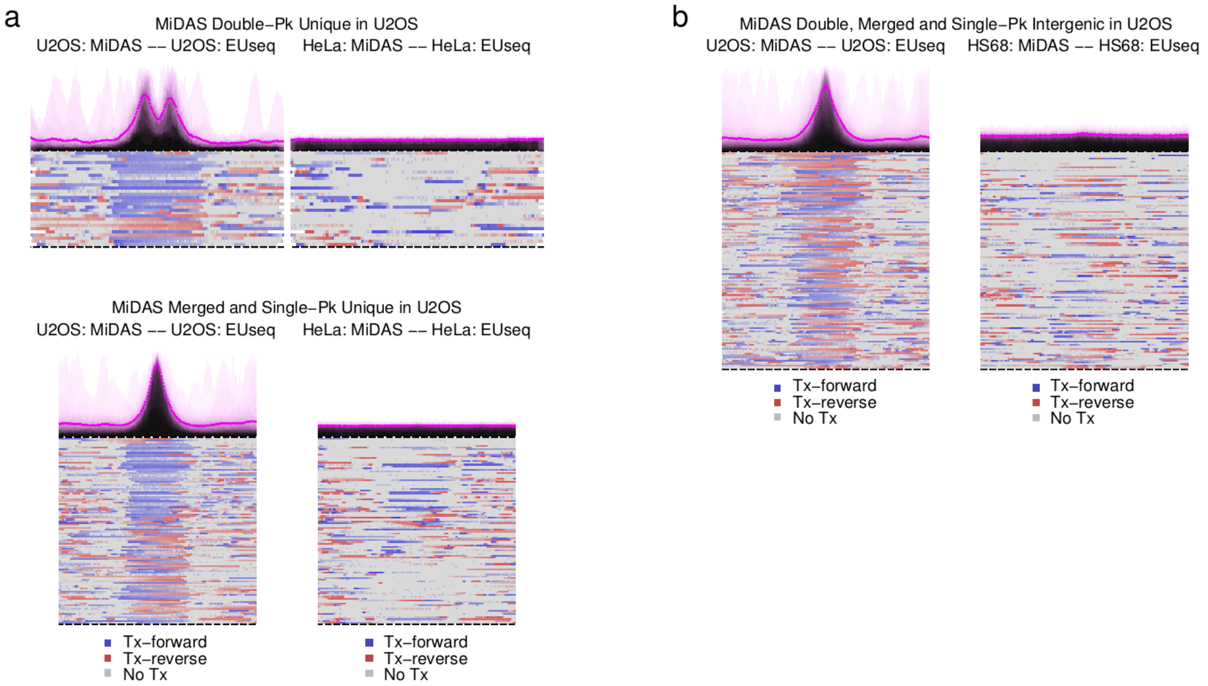

Supplementary Fig. S6. Correlation of MiDAS and transcriptional activity

a Nascent transcription heatmaps (U2OS, left panels; HeLa, right panels) for genomic regions that exhibit MiDAS in U2OS, but not in HeLa, cells. The genome-wide average MiDAS signal is shown above the heatmap plots. Span of genomic regions, 2.9 Mb (double-peak) or 2.3 Mb (merged and single-peak); Pk, peak; Tx, transcription.

b Nascent transcription heatmaps (U2OS, left panel; HS68, right panel) for all the intergenic genomic regions that exhibit MiDAS in U2OS cells. The genome-wide average MiDAS signal is shown above the heatmap plots. Span of genomic region, 2.4 Mb.
