# Supplementary material for: High-resolution mapping of mitotic DNA synthesis regions and common fragile sites in the human genome through direct sequencing
Source: Cell Res. 2020 Jun 19;30(11):997–1008. doi: 10.1038/s41422-020-0358-x (PMC7784693; doi:10.1038/s41422-020-0358-x)
Supplement: Supplementary file 7 — Supplementary Figure S7 [file 41422_2020_358_MOESM7_ESM.pdf]

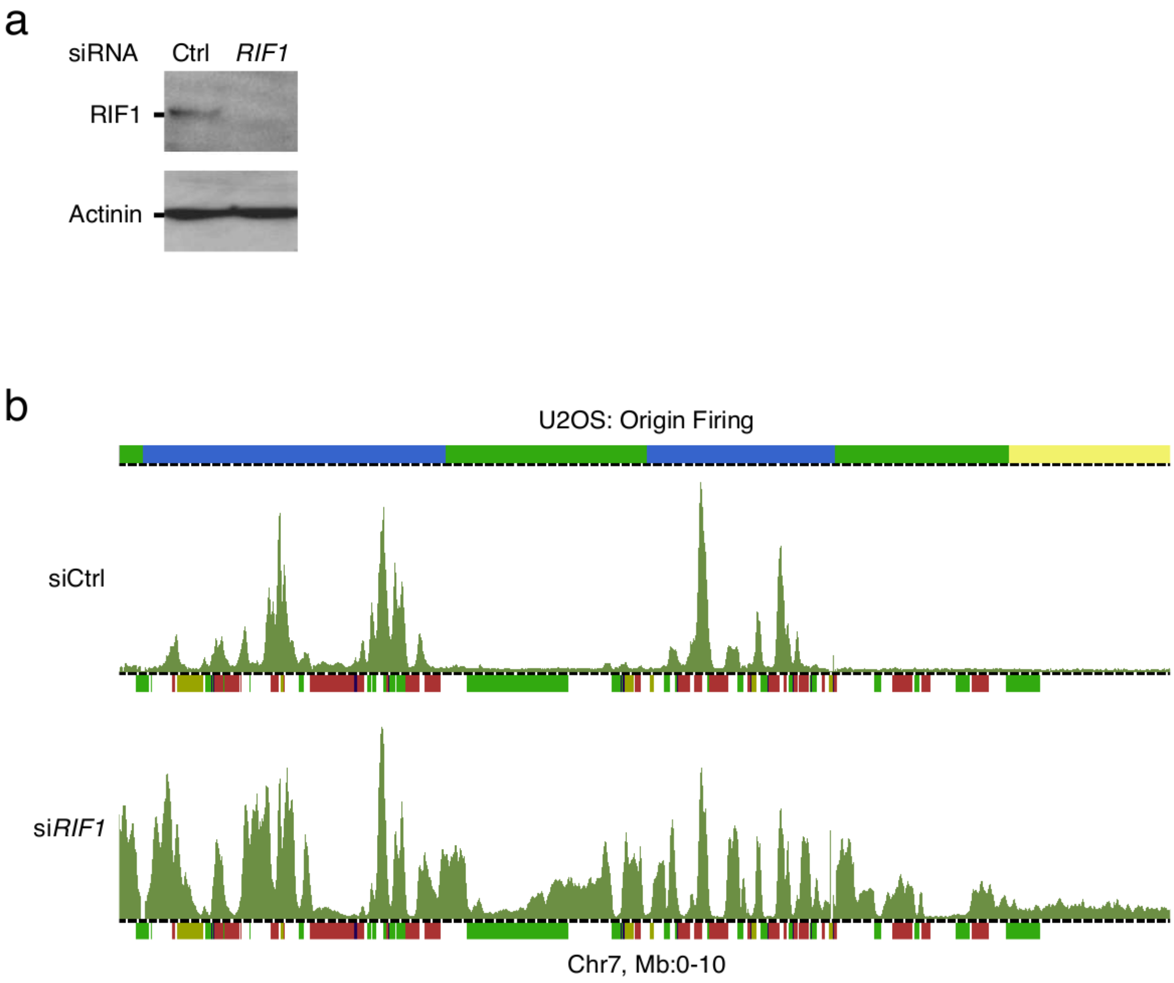

Supplementary Fig. S7. Efficiency of RIF1 depletion by siRNA and premature firing of DNA replication origins in mid S and late S replicating genomic domains

a Immunoblotting for RIF1 protein in lysates of cells transfected with siRNA targeting *RIF1* or a control (Ctrl) siRNA. Actinin was used as loading control.

b Origin firing profiles of control (Ctrl) and RIF1-depleted U2OS cells at the genomic region spanning the first 10 Mb of chromosome 7. The cells were synchronized by mitotic shake-off, released from the mitotic block and allowed to progress through G1 into S phase over 14 h in the presence of EdU, to label nascent DNA, and hydroxyurea, to limit fork progression. EdUseq signal is plotted as sigma values. Replication timing domains are shown at the top of the graphs: early S replicating domains (blue), mid S replicating domains (green), late S replicating domains (yellow). Bin resolution and ruler scale are as in Fig. 1b.
