# Supplementary material for: High-resolution mapping of mitotic DNA synthesis regions and common fragile sites in the human genome through direct sequencing
Source: Cell Res. 2020 Jun 19;30(11):997–1008. doi: 10.1038/s41422-020-0358-x (PMC7784693; doi:10.1038/s41422-020-0358-x)
Supplement: Supplementary file 8 — Supplementary Figure S8 [file 41422_2020_358_MOESM8_ESM.pdf]

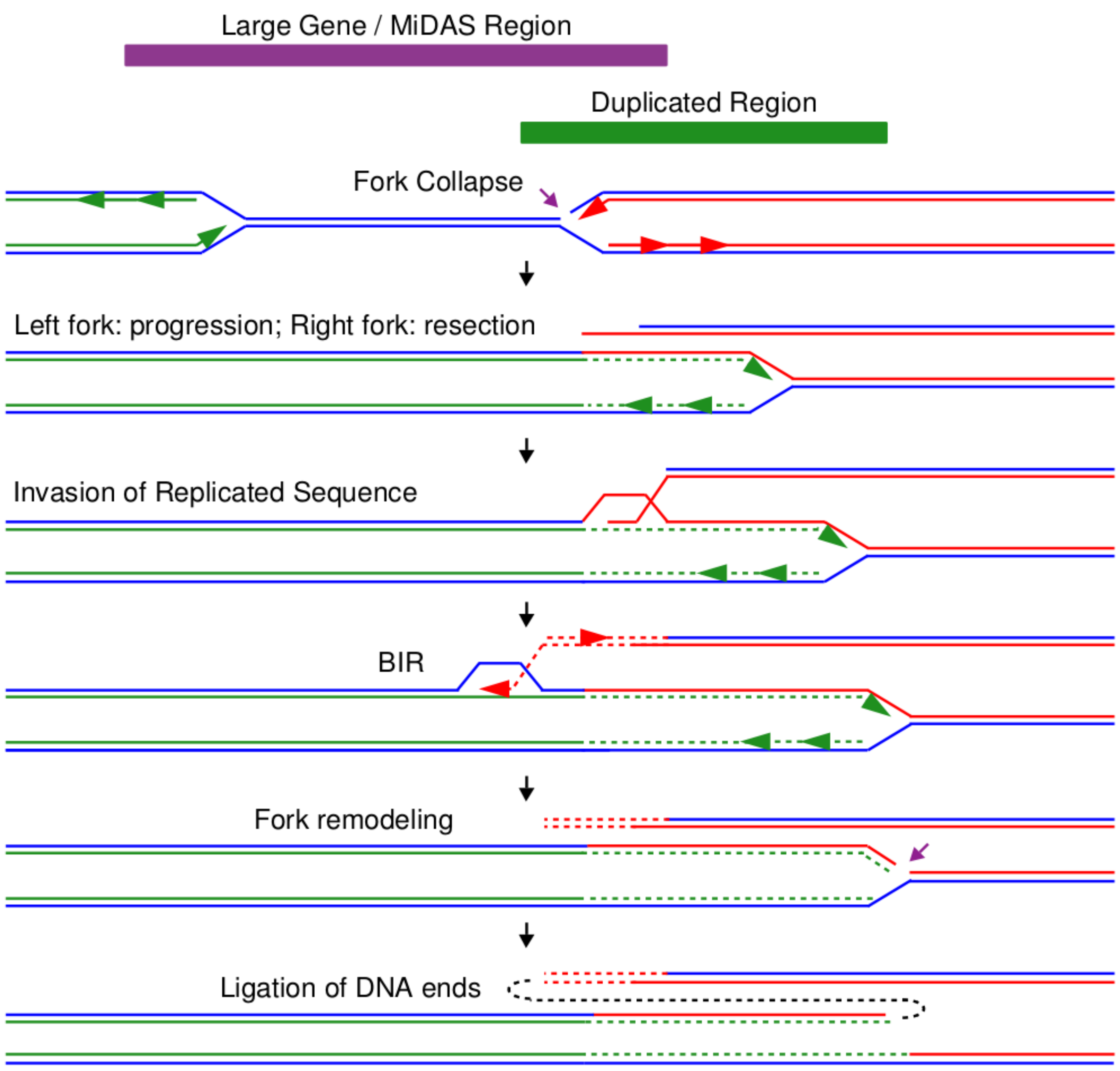

Supplementary Fig. S8. Model to explain the presence of genomic duplications around MIDAS regions

Collapse of a replication fork within a large gene in S phase will initiate repair by BIR. If a converging fork replicates the site of fork collapse, before the resected DNA end invades the DNA template molecule, then the region flanking the large gene will be duplicated due to it being replicated twice. The duplication would affect only one of the daughter molecules.
